# Coupled CRC 2D and ALI 3D Cultures Express Receptors of Emerging Viruses and Are More Suitable for the Study of Viral Infections Compared to Conventional Cell Lines

**DOI:** 10.1155/2020/2421689

**Published:** 2020-07-09

**Authors:** Siyu Xia, Jun Liu, Yan Yang, Ming Wu, Lina Ye, Si Chen, Tao Zhang, Zhihong Zeng, Kang Zhang, Kaihong Cai, Xiang Long, Wenbin Gao, Shisong Fang, Hui Li

**Affiliations:** ^1^State Key Laboratory of Virology/Institute of Medical Virology, School of Basic Medical Sciences, Wuhan University, Wuhan, Hubei 430071, China; ^2^Wuhan University Shenzhen Institute, Shenzhen, Guangdong 518057, China; ^3^Peking University Shenzhen Hospital, Shenzhen, 518036 Guangdong, China; ^4^Shenzhen Luohu People's Hospital, Shenzhen, 518001 Guangdong, China; ^5^Shenzhen Center for Disease Control and Prevention, Shenzhen, Guangdong 518055, China

## Abstract

Infections of emerging and reemerging viruses (SARS-CoVs, influenza H1N1, etc.) largely and globally affect human health. Animal models often fail to reflect a physiological status because of species tropism of virus infection. Conventional cell lines are usually genetically and phenotypically different from primary cells. Developing an *in vitro* physiological model to study the infection of emerging viruses will facilitate our understanding of virus-host cell interactions, thereby benefiting antiviral drug discovery. In the current work, we first established normal airway epithelial cells (upper and lower airway track) in 2D and 3D culture systems using conditional reprogramming (CR) and air-liquid interface (ALI) techniques. These long-term cultures maintained differentiation potential. More importantly, these cells express two types of influenza virus receptors, *α*2-6-Gal- and *α*2-3-Gal-linked sialic acids, and angiotensin-converting enzyme 2 (ACE2), a receptor for SARS-CoVs as well. These cells were permissive to the infection of pandemic influenza H1N1 (H1N1pdm). In contrast, the lung cancer cell line A549 and immortalized airway epithelial cells (16HBE) were not susceptible to H1N1 infection. A virus-induced cytopathic effect (CPE) on 2D CRC cultures developed in a time-dependent manner. The pathological effects were also readily observed spreading from the apical layer to the basal layer of the 3D ALI culture. This integrated 2D CRC and 3D ALI cultures provide a physiological and personalized *in vitro* model to study the infection of emerging viruses. This novel model can be used for studying virus biology and host response to viral infection and for antiviral drug discovery.

## 1. Introduction

Several major outbreaks of acute viral pneumonia caused by emerging viruses have greatly threatened public health since this century. In March 2009, a novel influenza virus emerged in Mexico and the United States. This virus was found to be a reassortant influenza H1N1 originated from multiple species-derived viruses. H1N1pdm gained the capacity to transmit in humans and quickly spread to more than 214 countries [1]. Thereafter, the H1N1 strain (pandemic influenza A, H1N1pdm) became a seasonal virus circulating over the world [2]. In February 2003, an outbreak of Severe Acute Respiratory Syndrome (SARS) was first reported in Guangdong Province of China. The pathogen was identified as SARS coronavirus (SARS-CoV) [3]. The SARS-CoV is an enveloped RNA virus and infected 8,096 cases and caused 774 deaths worldwide. In December 2019, a novel coronavirus, SARS-CoV2, caused an outbreak of acute pneumonia in Wuhan City of China [4]. On March 11, 2020, the World Health Organization (WHO) declared that SARS-CoV2 infection outbreak (COVID-19) can be characterized as a pandemic. These emerging viruses initially infect the respiratory epithelium and mainly transmit through the respiratory tract [1, 5]. Elucidating viral tropism in its primary target cells may help us understand the viral host-range, pathogenesis, and transmission.

Animals such as mice, pigs, quails, ferrets, chickens, and ducks have been used as influenza virus research models [2, 6–12]. Only ferrets and guinea pigs have been taken as the relevant surrogates for human influenza infection [5, 11]. Few animal models to study SARS-CoV include African green monkeys, macaques, and mice [13, 14]. However, animal models do not always reflect the human biology. The tissue tropism of viruses correlates with viral receptor expression on the target cells [15]. Human influenza viruses preferentially bind to *α*2-6-Gal-linked sialic acids while avian influenza viruses use *α*2-3-Gal-linked sialic acids as receptors [5, 16].

The fact that the human respiratory epithelium is the primary target cells for these emerging viruses brings us to the naïve model. Human respiratory tract-related models include human lung explants, primary airway epithelial cells, and immortalized airway epithelial cell lines or cancer cell lines. Human lung explants are not easily obtained and deteriorate rapidly *in vitro*. Cancer cell lines (e.g., A549) or oncogene-immortalized cells (e.g., SV40 large T-antigen immortalized human bronchial epithelial cells (16HBE)) can sustain their growth *in vitro*. However, the genetic background and phenotype of these cell lines have been changed. They can no longer represent the human airway epithelium with physiological function, for example, normal differentiation potential [17]. This is a major obstacle for studying virus-host cell interactions, thereby limiting antiviral drug discovery.

Human primary airway epithelial cells should be the solution. However, the culture of primary cells has been very challenging for decades. These primary epithelial cells can be cultured *in vitro* for limited few passages [18]. Most recently, two research teams used human airway organoids to assess infectivity of influenza virus [19, 20]. However, the inaccessibility of the apical surface to virus and inconvenient establishment of 3D organoids greatly limit its application. Therefore, the physiologically relevant, reproducible, and convenient models remain largely needed to study the tropism, pathogenesis, and transmission of emerging viruses. In terms of precision medicine, there is an unmet need for personalized host cells to study disease and host response to viral infection individually.

Recently, a new technique termed “conditional reprogramming” (CR) allows for the indefinite proliferation of human epithelial cells with no transduced viral or cellular genes [17, 21]. The CR method can be used to rapidly establish primary cell cultures and long-term expansion from fresh or cryopreserved tissue samples [21]. CR cells represent an adult stem-like state in the condition of irradiated mouse fibroblast cells and Rho kinase inhibitor [22]. CR cells maintain lineage differentiation potential *in vitro* [17, 22, 23]. The CR technique has been used to rapidly expand functional human respiratory epithelial cells with an unmet need by clinical transplantation [24]. In the present study, we first established primary human normal tracheal epithelial cells (HNTEC) and human normal bronchial epithelial cells (HNBEC) from two donors. These airway epithelial cells proliferated rapidly in defined culture conditions. HNTEC and HNBEC have the normal biological features and responses to stimuli. They express both types of influenza virus receptors and maintain tissue-specific differentiation potential. The coupled 2D and 3D differentiated cultures with human airway normal epithelial cells may provide an *in vitro* physiological model to assess the tropism and biology of influenza virus. Moreover, CR cells from a lower airway track expressed a higher level of viral receptor ACE2 mRNA compared to the upper airway CR cells, suggesting that these cells will also be suitable for studies of coronaviruses, such as SARS-CoVs.

## 2. Materials and Methods

### 2.1. Cell Culture

The normal tracheal or bronchial specimens were obtained by fiberoptic bronchoscopy of the patients. All patients gave their informed consent. The study was conducted in accordance with the Declaration of Helsinki, and the protocol was approved by the Ethics Committee of Peking University Shenzhen Hospital. Primary tissue preparation and culture procedures were performed according to the previous studies [21, 25, 26]. Briefly, the collected tissues were minced and digested into single-cell suspension. Primary tracheal or bronchial epithelial cells were cultured in a basic culture medium containing the Rho kinase inhibitor Y-27632 (PECBM, ImmorTech) with irradiated mouse fibroblasts or cultured in a primary epithelial cell culture medium containing the Rho kinase inhibitor Y-27632 (PECM, ImmorTech) without irradiated mouse fibroblasts at 37°C with 5% CO_2_. Mouse fibroblasts 3T3-J2 were irradiated as described previously [21] and obtained from Yongtech (Shenzhen). The irradiated mouse fibroblasts had their proliferation ceased and were easily removed by the initial 30-second trypsinization in a coculture system. 16HBE cells were obtained from the Shenzhen Center for Disease Control and Prevention. A549 cells (ATCC) and 16HBE cells were cultured in DMEM supplemented with 10% FBS. For 3D Matrigel culture, single cells were prepared into the differentiation medium (ImmorTech, China) with 2% Matrigel (BD Biosciences) [21]. For air-liquid interface (ALI) culture, 2 × 10^5^ cells were plated on 12 mm Millipore PCF polycarbonate inserts in PECBM. Two days later, the culture medium was replaced with ALI differentiation medium (CELLnTEC Advanced Cell Systems AG, Switzerland) according to the manufacturer's instructions. The cells were cultured at ALI for 19 days.

### 2.2. STR Analysis

Cell DNAs of HNTEC and HNBEC were extracted with the kit (TIANGEN). Short tandem repeat (STR) analyses were performed according to the previous studies [26].

### 2.3. Karyotype Analysis

A G-banded karyotype analysis was performed as described in previous studies [25, 27].

### 2.4. Virus Infection

Pandemic influenza H1N1 (H1N1pdm) was previously isolated by the Shenzhen Center for Disease Control and Prevention [28, 29]. The H1N1pdm virus was propagated in MDCK cells (Madin-Darby Canine Kidney). At 72 hours postinfection (hpi), the culture medium without cells was harvested, aliquoted, and stored at -80°C. Virus titration was determined by a plaque assay [19]. For virus infection, 2D monolayer cultured cells were inoculated with viruses at the multiplicity of infection (MOI) of 0.001, and trypsin (TPCK) was added [28]. ALI 3D cultures were differentiated for 19 days and then inoculated with viruses at MOI of 0.01 at the apical layer. Virus inoculums were removed after 1 hr adsorption. The cultures were rinsed with PBS for 3 times and replenished with fresh medium. At the indicated time points, cells in 2D or 3D cultures were collected or fixed.

### 2.5. Western Blotting Analysis

Cells in 2D cultures were first incubated with or without actinomycin D for 24 hours before harvesting. The western blotting assay was performed according to the previous study [27].

### 2.6. Immunofluorescent Assay

Cells in 2D or 3D cultures were treated with 4% (*w*/*v*) paraformaldehyde and then permeabilized with 0.5% Triton X-100. The first blotting antibodies (mouse antibody against mucin 5A, Abcam; mouse antibody against p63, Abcam; mouse antibody against cytokeratin 14, Abcam; and mouse antibody against influenza A virus nucleoprotein, Abcam ab20343) were incubated according to the manufacturer's procedures and previous studies [23, 25, 27]. 0.5 *μ*g/ml DAPI (D3571) was used to stain the nuclei. All images were captured by the Leica DM4000B fluorescence microscope.

### 2.7. DAB (3,3′Diaminobenzidine) Staining

The DAB staining was carried out according to the previous study [27], using a commercial kit called DAB Detection Kit (EliVision Super DAB, Maixin Biotech). The primary antibodies used were as follows: mouse antibody against p63 (ab735, Abcam), rabbit antibody against CK5 (ab53121, Abcam), and mouse antibody against CK14 (sc-23878, Santa Cruz Biotechnology).

### 2.8. Histochemistry

Tissues and 3D cultures were first treated with 4% (*w*/*v*) paraformaldehyde and paraffin-embedded according to the standard histological procedure [27, 30]. The tissue sections were stained with H&E (Hematoxylin and Eosin) (Zhongshan Golden Bridge Company). Histology images were analyzed and captured with the EVOS flat screen microscope (Life Technologies).

### 2.9. Soft Agar Assay

Soft agar assays with 4 × 10^4^ cells in 0.3% low-melting point agarose were performed according to a previous study [26]. Colony images were analyzed and captured with the EVOS flat screen microscope (Life Technologies).

### 2.10. Lectin Immunofluorescence Staining

Cells in 2D or 3D cultures were fixed and permeabilized as described above, then labeled with the biotinylated lectins (20 *μ*g/ml, SNA, Vector Laboratories, Burlingame, CA, B-1305; 20 *μ*g/ml, MAA-I, Vector Laboratories, Burlingame, CA, B-1265; and 20 *μ*g/ml, MAA-II, Vector Laboratories, Burlingame, CA, B-1315), and then detected with fluorescein avidin DCS (A-2011, Vector Laboratories, Burlingame, CA) at 37°C for an hour in the dark. 0.5 *μ*g/ml DAPI (D3571) was used to stain the cell nuclei. The staining was analyzed and captured with the Leica DM4000B fluorescence microscope.

### 2.11. RNA Extraction and Real-Time RT-PCR

Total cellular RNAs were extracted using the TRIzol Reagent (Invitrogen), and real-time RT-PCR was performed as described previously [27]. The primer sequences for the detection of ACE2 mRNA were 5′-CATTGGAGCAAGTGTTGGATCTT-′3 (sense) and 5′-GAGCTAATG-CATGCCATTCTCA-3′ (antisense) located from 2864 to 2886 bp (exon 18) and 2950 to 2971 bp (exon 18), respectively, and amplify a 107 bp region of the mRNA.

## 3. Results

### 3.1. Establishment of Long-Term Stable Cultures of Normal Airway Epithelial Cells

**Figure 1:**
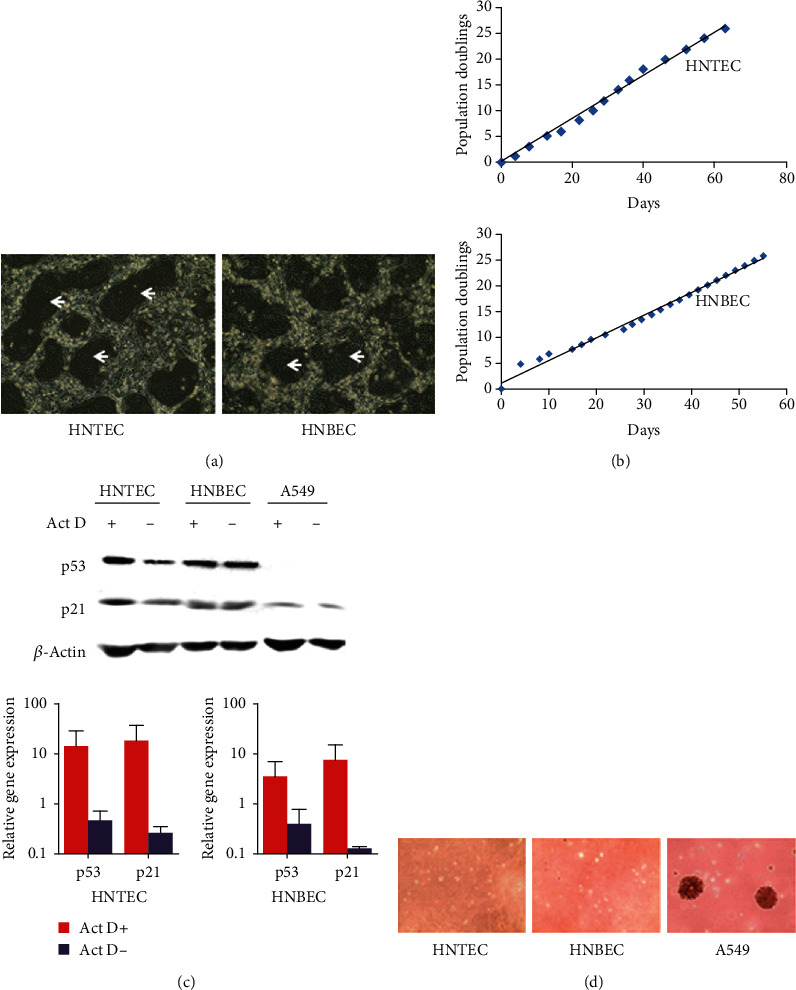
Generation and characterization of human airway normal epithelial cells. (a) The morphology of the primary HNTEC and HNBEC. HNTEC and HNBEC were cocultured and grew rapidly. The colonies of epithelial cells were observed at 2 days after the initial cell isolation. The arrows indicate the colonies of epithelial cells. Magnification 10x. (b) Growth curves of two strains of airway epithelial cells, HNTEC and HNBEC. (c) Cell response to DNA damage. The cells were treated with 0.5 nM actinomycin D for 24 hours. Expression of p53 and p21 was measured with western blotting and quantitative RT-PCR. (d) The colony formation of HNTEC and HNBEC. HNTEC, HNBEC, and A549 cells were used in soft agar assays. Only the lung cancer line A549 formed colonies in soft agar.

**Figure 2:**
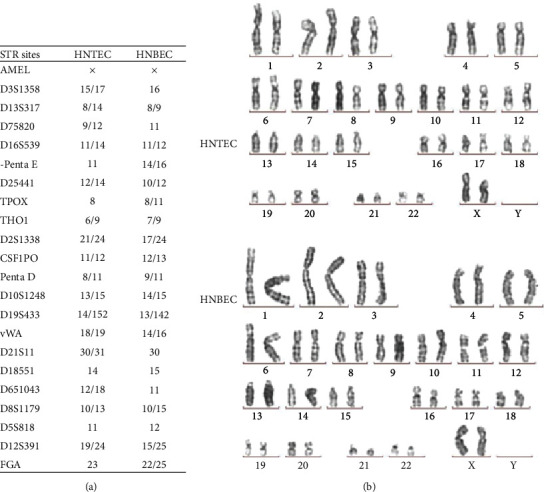
The STR and karyotype analysis of airway normal epithelial cells. (a) Short tandem repeat (STR) analysis of HNTEC and HNBEC. STR patterns from HNTEC and HNBEC did not match any other cell lines registered or published before. (b) Normal karyotypes of HNTEC and HNBEC.

It has been shown that the CR technique can efficiently establish individual-derived primary cell cultures without gene manipulation [21]. The establishment of the primary CR cell cultures is rapid. Consistent with a previous study, the colonies of human airway epithelial cells could be observed within 2 days of cell isolation. Morphologies of HNTEC and HNBEC cocultured with irradiated mouse fibroblast cells (feeder) are shown in Figure 1(a). The CR technique is using feeder cells and Rho kinase inhibitor (Y-27632) to induce indefinite cell proliferation *in vitro* [21, 24]. Growth curves of HNTEC and HNBEC are shown in Figure 1(b). The short tandem repeat (STR) analysis verified two new cultures from human tissues (Figure 2(a)). HNTEC and HNBEC possess a structurally and numerically normal karyotype with 46,XX (Figure 2(b)). These cells are karyotype-stable. HNTEC and HNBEC did not form cell colonies and existed as single cells or cell debris in soft agar culture for 30 days (Figure 1(d)). In contrast, the cancer cell line A549 formed anchorage-independent colonies (Figure 1(d)). These results indicated that HNTEC and HNBEC are nontumorigenic. To investigate whether HNTEC and HNBEC have the intact p53-mediated growth-related pathways and normal function to respond to the DNA damage, the cells were treated with actinomycin D (Act D).  Figure 1(c) shows that p53 level was upregulated in cells with Act D treatment and the downstream effector p21 was also increased compared to untreated cells (Figure 1(c)). However, in A549 cells treated with Act D, p53 protein level was not induced and neither p21 was upregulated compared to untreated A549 cells. These results indicated that airway epithelial cells normally responded to the DNA damage. Taken together, these data indicated that we established the stable long-term cultures of airway normal epithelial cells from personalized tissues.

### 3.2. Tissue-Specific Differentiation Potential of Human Airway Normal Epithelial Cells

**Figure 3:**
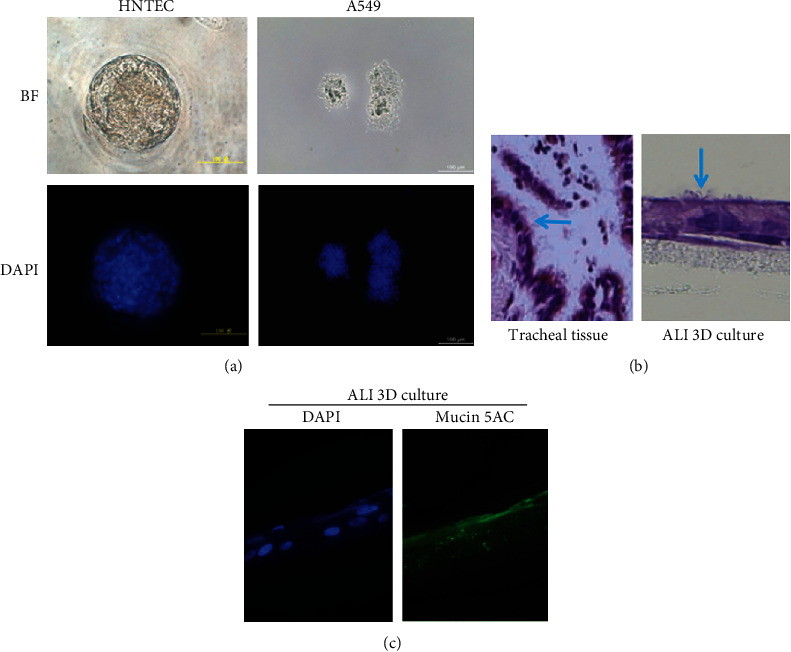
The tissue-specific differentiation potential of airway normal epithelial cells. (a) Matrigel 3D cultures of HNTEC. HNTEC and A549 cells were cultured in 5% Matrigel for 7 days. Matrigel 3D cultures were stained with 0.5 *μ*g/ml DAPI and analyzed with fluorescence microscopy. Scale bar, 200 *μ*m. (b) Histology comparison of the original tracheal tissues and 3D ALI culture of HNTEC. HNTEC were cultured in 3D ALI for 19 days. Magnification 40x. (c) Expression of mucin 5AC in HNTEC-derived ALI 3D culture. 3D ALI cultures were fixed, permeabilized, and labeled with the primary antibody against mucin 5AC. The expression of mucin 5AC was measured by immunofluorescence protocols. The cellular nuclei were stained with 0.5 *μ*g/ml DAPI. Magnification 40x.

The differential potential of normal cells is important for their physiological function [31]. Influenza virus replication is also greatly influenced by the state of differentiation [32]. CR cells possess the ability to differentiate normally [22]. The Matrigel basement membrane matrix is an important regulatory factor to maintain homeostasis of cells and tissue morphogenesis [33]. We performed Matrigel three-dimensional (3D) culture to evaluate the differential potential of these individualized airway normal epithelial cells.  Figure 3(a) shows that HNTEC formed well-organized and polarized spheres in Matrigel 3D culture. In contrast, lung cancer A549 cells generated disorganized and nonpolar aggregates. 3D ALI culture was also used to evaluate the differentiation potential of airway epithelial cells. We did compare the histology structures between the tracheal tissue and 3D ALI cultures. HNTEC-derived 3D ALI cultures formed a pseudostratified epithelium with cilia; these were similar to the original tracheal tissue (Figure 3(b)). The expression mucin 5AC in HNTEC-derived ALI 3D culture indicated the secretory goblet cells (Figure 3(c)). Thus, CR airway epithelial cells possess normal differentiation potential *in vitro*.

Conditionally reprogrammed epithelial cells maintain an adult stem-like state [22]. Next, we analyzed the tissue-specific marker of HNTEC by immunofluorescence and DAB staining. Two-dimensional (2D) cultured HNTEC express the epithelium-specific marker cytokeratin 14 (CK14) and airway basal cell marker cytokeratin 5 (CK5) the same as the tracheal tissue (Figures S1 and S2). Besides, HNTEC also express the stemness marker p63 (Figure S2). Under the differentiation condition, HNTEC-derived Matrigel or ALI 3D culture expresses CK14 and CK5 similar to its original tracheal tissue (Figure S2b and S2(c)). Taken together, our results demonstrated that airway normal CR epithelial cells maintained the tissue-specific differentiation potential. Human airway normal epithelial cell-derived 3D culture could mimic the original structure of the tracheal/bronchial epithelium morphologically and physiologically.

### 3.3. Expression of Virus Receptors on Airway Normal Epithelial Cells and HNTEC-Derived Differentiated 3D Cultures

**Figure 4:**
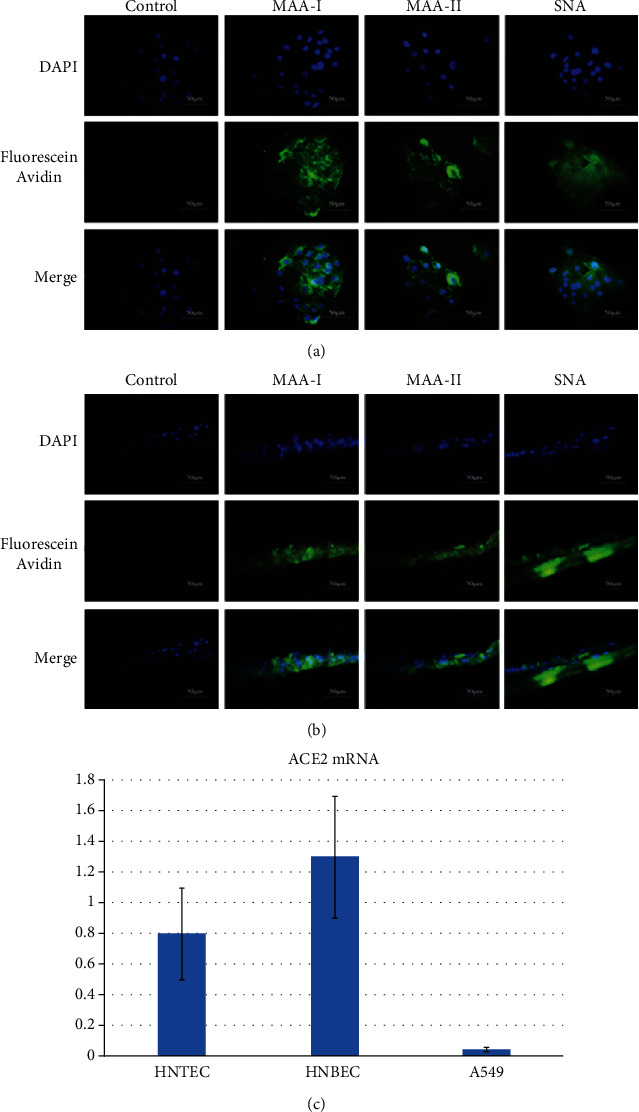
Influenza virus sialic acid receptor detection on 2D and 3D ALI cultures of HNTEC. (a) Influenza virus sialic acid receptor detection on 2D cultures of HNTEC. HNTEC were seeded in 12-well plates cultured at an appropriate density, then fixed and permeabilized as described above, and labeled with the biotinylated lectins SNA, MAA-I, and MAA-II and PBS (as a blank control), respectively. These three lectin markers were measured by immunofluorescence protocols. The cellular nuclei were stained with DAPI. The lectins were stained with fluorescein avidin DCS. Magnification 40x. Scale bar, 50 *μ*m. (b) Influenza virus sialic acid receptor detection on 3D ALI cultures of HNTEC. ALI 3D cultures of HNTEC were fixed, permeabilized, and labeled with the biotinylated lectins SNA, MAA-I, and MAA-II, respectively. The nuclei were stained with DAPI. The lectins were stained with fluorescein avidin DCS. Magnification 40x. Scale bar, 50 *μ*m. (c) Expression of ACE2 mRNA in HNTEC and HNBEC. Total cellular RNAs were extracted, and real-time RT-PCR was performed to detect the expression of ACE2 mRNA. The cancer cell line A549 was used as control cells.

Sialic acid alpha 2,3-galactose (Sia *α*2-3-gal) and sialic acid alpha 2,6-galactose (Sia *α*2,6-gal) are two classic types of influenza virus receptors [34]. Human influenza viruses preferentially bind to *α*2-6-Gal-linked sialic acids while avian influenza viruses exhibit a preference for *α*2-3-Gal-linked sialic acids [5, 16]. The lectin binding assay was usually used to detect influenza virus receptors. *Sambucus nigra* agglutinin (SNA) can specifically recognize *α*2-6-linked sialic acids, while *Maackia amurensis* agglutinin- (MAA-) I recognizes *α*2,3 N-linked sialic acids and MAA-II recognizes *α*2,3 O-linked sialic acids [5]. The expression and distribution of the sialic acid receptors have a major influence in cells' sensitivity or permissiveness to human or avian influenza viruses [32]. Therefore, we evaluated the expression of sialic acid receptors by the lectin binding assay with immunofluorescence.  Figure 4(a) shows that MAA-I, MAA-II, and SNA could bind to HNTEC. MAA-I exhibited the strongest binding to HNTEC while SNA and MAA-II bound weakly (Figure 4(a)). The results suggested that these two types of receptors for influenza virus infection were expressed in 2D cultured HNTEC. The monolayer undifferentiated HNTEC expressed more avian influenza virus receptors. We also observed that SNA, MAA-I, and MAA-II bound to the basal and apical layers of HNTEC-derived 3D ALI cultures (Figure 4(b)). The abundance of SNA binding was the strongest. MAA-II binding was much weaker than that of MAA-I and SNA. The results indicated that well-differentiated HNTEC-derived 3D cultures expressed more human influenza virus receptors similar to that in tracheal tissue [32]. Since other emerging or reemerging viruses may also infect airway epithelial cells, for example, SARS-CoV2, which is causing a COVID-19 outbreak in Wuhan, China, we also asked whether these airway epithelial cells express ACE2 (a potential receptor for SARS-CoV2 infection). Interestingly, both HNTEC and HNBEC expressed ACE2 mRNA although the lower airway epithelial cells (HNBEC) expressed relatively higher levels of ACE2 mRNA (Figure 4(c)).

### 3.4. Influenza Virus Infection of 2D and Differentiated 3D Culture of Airway Epithelial Cells

**Figure 5:**
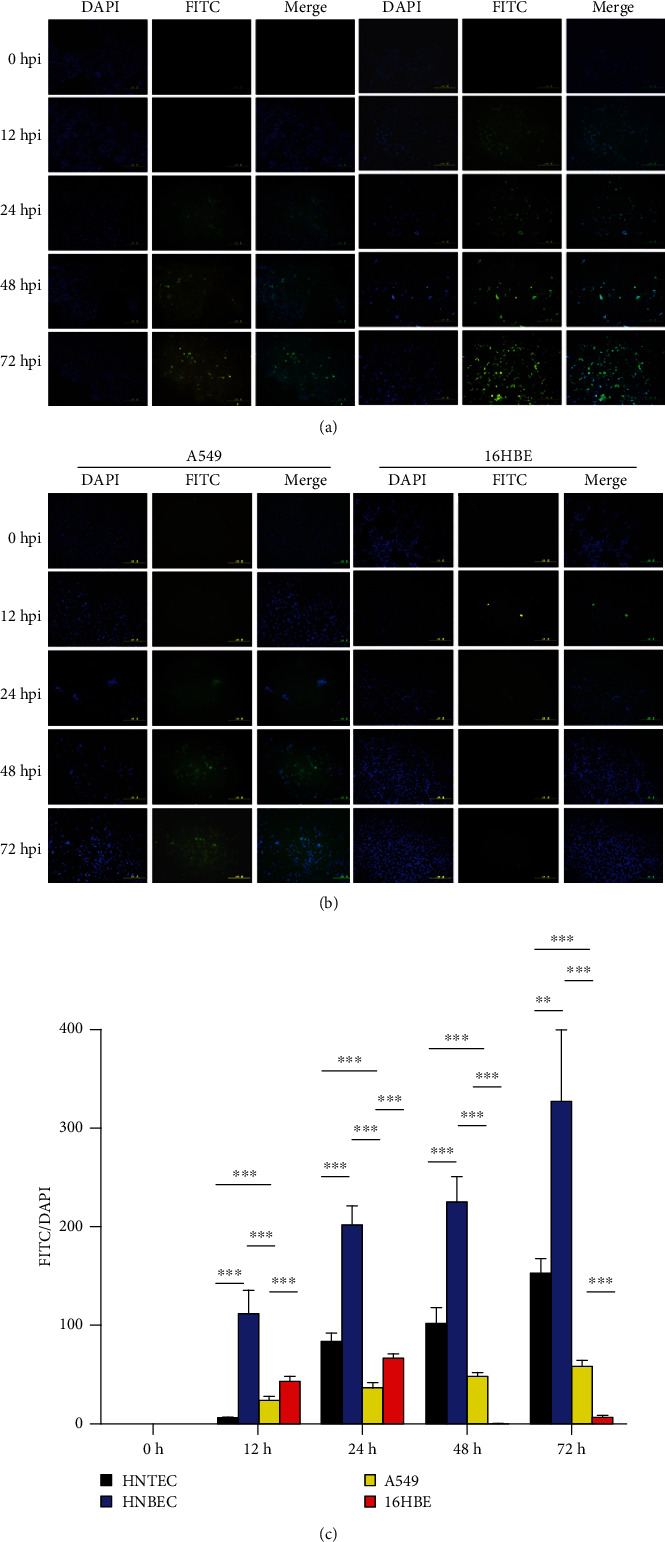
H1N1 replication in 2D airway normal epithelial cells. (a) Expression of influenza A virus nucleoprotein in HNTEC and HNBEC. Cells were seeded at 5 × 10^5^ per well in 6-well plates 24 h before inoculation of the H1N1pdm virus. The 2D monolayer cultured cells were inoculated with viruses at multiplicity of infection (MOI) of 0.001. Infected cells were harvested at the indicated time points. Then, cells were fixed with 4% (*w*/*v*) paraformaldehyde and permeabilized with Triton X-100 and then labeled with the antibody against anti-influenza A virus nucleoprotein. Expression of influenza A virus nucleoprotein was detected by the immunofluorescence assay. The nuclear staining was used with 0.5 *μ*g/ml DAPI. Magnification 20x. Scale bar, 100 *μ*m. (b) Expression of influenza A virus nucleoprotein in A549 and 16HBE cells. (c) Quantitation of influenza A virus nucleoprotein-positive cells. The experiment was repeated for three times. Averaged data are means ± SE mean. Differences between cells were assessed using the unpaired *t*-test (*n* ≥ 3; ^∗^*p* < 0.05, ^∗∗^*p* < 0.01, and ^∗∗∗^*p* < 0.001).

**Figure 6:**
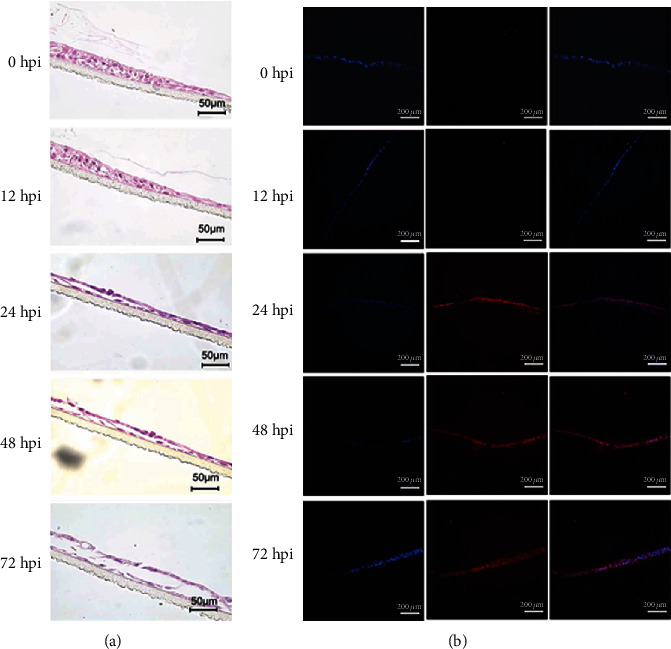
H1N1 infection of 3D ALI cultures of HNTEC. (a) Morphology of 3D ALI cultures of HNTEC infected by H1N1pdm. HNTEC were cultured in 3D ALI as indicated in days and then inoculated with viruses at MOI of 0.01 at the apical layer. Virus inoculums were removed, after 1 hr adsorption. The cultures were rinsed with PBS for 3 times and replenished with fresh medium. At the indicated time points, 3D cultures were harvested. The 3D ALI cultures were then fixed, paraffin-embedded, and sectioned. Histology was captured under the microscope. Scale bar, 50 *μ*m. (b) Expression of influenza A virus nucleoprotein in 3D ALI cultures after H1N1pdm infection. 3D ALI cultures with H1N1pdm were stained with the antibody against anti-influenza A virus nucleoprotein. Expression of influenza A virus nucleoprotein detected by immunofluorescence protocols. The nuclear staining was used with 0.5 *μ*g/ml DAPI. Scale bar, 100 *μ*m.

Our results demonstrated that human normal airway epithelial cells and corresponding differentiated 3D cultures express both types of influenza virus receptors (Figure 4). Next, we investigated their susceptibility to viral infection. 2D monolayer cultured HNTEC and HNBEC could be infected by the influenza A virus H1N1 (H1N1pdm) (Figure S3). As early as 12 hr postinfection, we could observe a cytopathic effect (CPE) in human normal airway epithelial cells. The CPE developed more quickly in HNBEC than in HNTEC. The immunofluorescence staining with influenza A virus nucleoprotein indicated the appearance (entry) of viruses at 12 hpi in HNBEC and 24 hpi in HNTEC (Figure 5(a)). The results showed that HNBEC are more susceptible than HNTEC to H1N1pdm virus infection (Figure 5(c)). These might be due to difference of donor individuals or anatomic sites or the expression of the viral receptor. The control cancer cell line A549 cells could be infected by H1N1pdm virus, but CPE appeared at 24 hpi and virus replication increased limitedly up to 72 hpi (Figures 5(b) and 5(c) and Figure S3). The other control cell line 16HBE (oncogene-immortalized human bronchial epithelial) cells were not susceptible enough to H1N1pdm virus infection and replication, as shown by the CPE observation and immunofluorescence assay (Figures 5(b) and 5(c) and Figure S3). HNTEC-derived 3D ALI cultures were used to investigate the susceptibility of well-differentiated airway epithelial cells to the H1N1pdm virus. The H1N1pdm virus was inoculated to the apical layer of differentiated 3D ALI cultures as described in Materials and Methods. The CPE was examined by histology (Figure 6(a)). At 12 hpi, the HNTEC-derived 3D culture became disorganized. At 24 hpi and 48 hpi, the pseudostratified epithelium structure was destructed gradually. At 72 hpi, the apical layer detached from the basal layer completely, and the 3D structure was lost. The immunofluorescence staining with influenza A virus nucleoprotein indicated the replication of H1N1pdm virus starting at 24 hpi (Figure 6(b)). The H1N1pdm virus propagated till the 72 hpi observed time course.

## 4. Discussion

The human respiratory epithelium is the primary target and host cell for the influenza virus. The tropism and replication competence of influenza viruses in the respiratory tract have consequences for viral transmission and pathogenesis [35]. Many efforts have been put to establish the physiologically relevant experimental model to study the virology and pathogenesis of influenza viruses in humans. Transformed or gene-immortalized human cell lines are being ruled out of the physiological models because of their important differences from the corresponding tissues *in vivo* [17]. Human respiratory tract explants or *ex vivo* cultures were first adopted in the 1960s and are still used for influenza virus research [32, 36]. But the routine availability and maintenance of tissue viability of respiratory tract explants are unsolved issues. Primary human airway epithelial cells have been widely used although these cells usually undergo senescence after a few passages in dishes [5, 32]. The tropism and replication of the influenza virus are greatly influenced by the differentiation state of the airway epithelium [19, 32]. Primary airway cells are considered to be used with differentiation in air-liquid interface (ALI) cultures or organoids (alternatively named Matrigel 3D cultures). The presence of mucin-secreting goblet cells and cilia in the pseudostratified epithelium is the key marker for a well-differentiated epithelium [19, 32]. CR cells maintain lineage differentiation potential [17, 22, 23]. Under the differentiation conditions (withdrawal of feeder cells and ROCK inhibitors), CR cells differentiate automatically *in vitro* and form the 3D “miniorgan” which recaptures the structure and physiological function of the original tissue [17, 22]. Our results demonstrated that HNTEC-derived ALI 3D cultures formed stratified epithelial structures with cilia compared to the original tracheal tissue and also expressed mucin 5AC. Moreover, HNTEC-derived Matrigel or ALI 3D cultures express CK14 and CK5, similar to their original tracheal tissue. CR airway epithelial-derived 3D culture formed a well-differentiated airway epithelium which mimics an *in vivo* airway epithelium structure.

Recently, there are reports using human airway 3D organoids and derived 2D monolayer culture to identify infective influenza viruses [19, 20]. The procedure of organoid culture is to embed isolated primary cells into Matrigel. Matrigel contains an extracellular matrix rich in laminin and maintains normal homeostasis and tissue morphology [33]. Since Matrigel is the extract from sarcoma cells, different brands and even batches of product influence the morphology and success rate of organoids. Another limitation of organoids is the inaccessibility of the apical layer to the virus inoculum. To overcome this weakness, 3D organoids have to be digested and seeded in inserts (Transwell) and cultured in optimized medium [19]. The primary cells embedded in Matrigel could hardly be characterized. The entire procedure of establishment of 3D organoids and 2D monolayer of airway cultures is actually not convenient or reproducible and biologically relevant. In contrast, primary airway normal epithelial cells can be established by the CR method sufficiently and expanded rapidly. Since these CR cells are continuous cell culture, we can clearly define their genetic background and biological characteristics. CR airway epithelial cells maintain both advantages of immortalized cell lines and primary cultures. These CR airway epithelial cells also meet the need for the selection of relevant phenotype individual for emerging virus research. In the future study, more samples from different anatomic sites derived from the same individual may be needed to assess the difference in virus sensitivity of HNTEC and HNBEC.

The expression and distribution of the viral receptor are another critical issue to evaluate the physiologically related model for respiratory tract virus infection. It has been reported that well-differentiated human bronchial epithelial cells exhibited strong binding with MAA-I (*α*2-3 N-linked Gal, a receptor for the avian influenza virus) and SNA (*α*2-6-Gal, a receptor for the human influenza virus) and weak binding with MAA-II (*α*2-3 O-linked Gal) [5, 32]. Others reported that the human influenza virus may replicate more sufficiently than the avian influenza virus in differentiated human airway epithelial cells [37]. Our results are in accordance with these findings that SNA exhibited the strongest binding to HNTEC-derived ALI 3D cultures and H1N1pdm replicated efficiently in well-differentiated HNTEC-derived 3D cultures. We also investigated the expression differences of influenza virus receptors in 2D cultured airway epithelial cells. Although 2D monolayer cultured airway epithelial cells expressed more avian influenza virus receptors, the human influenza virus could replicate efficiently in 2D undifferentiated airway epithelial cells. HNTEC and HNBEC isolated from two donors demonstrated different sensitivity to H1N1 infection; these might be due to the difference of donor individuals or anatomic sites or the expression of the viral receptor. Cancer cell line or gene-immortalized cells exhibited none or very low sensitivity to viral infection. These data indicated that CR airway epithelial cells could serve as a reliable physiologically relevant experimental system for studies of emerging or reemerging viruses.

**Figure 7:**
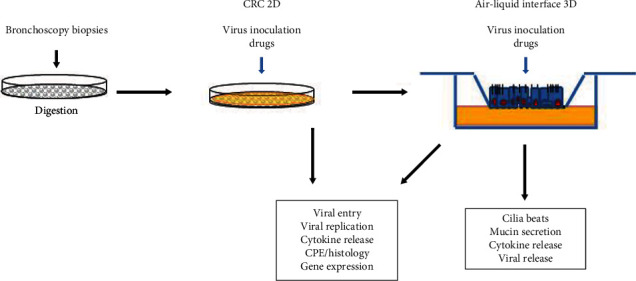
Illustration of coupled CRC 2D and ALI 3D cultures for the study of viral infection.

Our current study largely focused on the establishment of an *in vitro* CRC 2D and ALI 3D integrated model and its feasibility for virus infection (Figure 7). Further and collaborative investigation on the virology, pathogenesis, and antiviral drug discovery will be followed up in later publications.

## 5. Conclusion

We established 2D and 3D cultures from the normal human airway epithelium, which expressed receptors of emerging viruses and are suitable for viral infection; this combination of cultures provides a physiological and personalized *in vitro* model to study the infection of emerging viruses. This novel model can be used for virus biology, host response to viral infection, and antiviral drug discovery.

## Data Availability

The data used to support the findings of this study are included within the article and Supplementary Materials.

## Supplementary Materials

Supplementary Materials Figure S1: expression of tissue-specific markers in human tracheal tissue. (A) Immunohistochemical staining of human tracheal tissue. Tissue was fixed with 4% paraformaldehyde (wt/vol), paraffin-embedded, and sectioned detected by immunohistochemical staining with the specific antibodies against CK5, laminin, DSG-1, and E-cadherin. Scale bar, 50 *μ*m. (B) Immunofluorescence staining of human tracheal tissue. Tissue was fixed with 4% paraformaldehyde (*w*/*v*), permeabilized with 0.5% Triton X-100, and labeled with the primary antibodies against CK14. The expression of CK14 was detected by the immunofluorescence assay. The nuclei were stained with 0.5 *μ*g/ml DAPI. Magnification 20x. Figure S2: expression of tissue-specific markers in HNTEC and derived 3D cultures. (A) Expression of tissue-specific markers in HNTEC. HNTEC were cultured on the sterile glass coverslips at an appropriate density and fixed in 4% (*w*/*v*) paraformaldehyde, permeabilized with 0.5% Triton X-100, and labeled with the primary antibodies against CK14, CK5, and p63, respectively. These three protein markers were detected by the immunofluorescence assay. The nuclei were stained with 0.5 *μ*g/ml DAPI. The proteins were stained with the secondary antibody goat-anti-mouse IgG-488. Scale bar, 100 *μ*m. (B) Expression of tissue-specific markers in Matrigel 3D cultures of HNTEC. Single-cell suspension of HNTEC and A549 cells was cultured in medium containing 5% Matrigel for 7 days. Matrigel 3D cultures were fixed with 4% paraformaldehyde (*w*/*v*), permeabilized with 0.5% Triton X-100, and labeled with the primary antibodies against CK14, CK5, and p63. Scale bar, 100 *μ*m. (C) Expression of tissue-specific markers in ALI 3D cultures of HNTEC. HNTEC were cultured in an air-liquid interface (ALI) for 19 days and fixed with 4% paraformaldehyde (wt/vol), paraffin-embedded, and sectioned using standard histological procedures. Expression of tissue-specific markers was detected by immunohistochemical staining with antibodies against CK14, CK5, and p63. Scale bar, 50 *μ*m. Figure S3: H1N1 infection of 2D cultured airway normal epithelial cells. HNTEC and HNBEC were seeded at 5 × 10^5^ per well in 6-well plates 24 hr before inoculation of the H1N1pdm virus. A549 and 16HBE cells were used as control cells. Morphology of cells was photographed at the indicated time points. Magnification 20x. Scale bar, 100 *μ*m.
