# Supplementary material for: Coupled CRC 2D and ALI 3D Cultures Express Receptors of Emerging Viruses and Are More Suitable for the Study of Viral Infections Compared to Conventional Cell Lines
Source: Stem Cells Int. 2020 Jul 9;2020:2421689. doi: 10.1155/2020/2421689 (PMC7368225; doi:10.1155/2020/2421689)
Supplement: Supplementary Materials — Figure S1: expression of tissue-specific markers in human tracheal tissue. (A) Immunohistochemical staining of human tracheal tissue. Tissue was fixed with 4% paraformaldehyde (wt/vol), paraffin-embedded, and sectioned detected by immunohistochemical staining with the specific antibodies against CK5, laminin, DSG-1, and E-cadherin. Scale bar, 50 μm. (B) Immunofluorescence staining of human tracheal tissue. Tissue was fixed with 4% paraformaldehyde (w/v), permeabilized with 0.5% Triton X-100, and labeled with the primary antibodies against CK14. The expression of CK14 was detected by the immunofluorescence assay. The nuclei were stained with 0.5 μg/ml DAPI. Magnification 20x. Figure S2: expression of tissue-specific markers in HNTEC and derived 3D cultures. (A) Expression of tissue-specific markers in HNTEC. HNTEC were cultured on the sterile glass coverslips at an appropriate density and fixed in 4% (w/v) paraformaldehyde, permeabilized with 0.5% Triton X-100, and labeled with the primary antibodies against CK14, CK5, and p63, respectively. These three protein markers were detected by the immunofluorescence assay. The nuclei were stained with 0.5 μg/ml DAPI. The proteins were stained with the secondary antibody goat-anti-mouse IgG-488. Scale bar, 100 μm. (B) Expression of tissue-specific markers in Matrigel 3D cultures of HNTEC. Single-cell suspension of HNTEC and A549 cells was cultured in medium containing 5% Matrigel for 7 days. Matrigel 3D cultures were fixed with 4% paraformaldehyde (w/v), permeabilized with 0.5% Triton X-100, and labeled with the primary antibodies against CK14, CK5, and p63. Scale bar, 100 μm. (C) Expression of tissue-specific markers in ALI 3D cultures of HNTEC. HNTEC were cultured in an air-liquid interface (ALI) for 19 days and fixed with 4% paraformaldehyde (wt/vol), paraffin-embedded, and sectioned using standard histological procedures. Expression of tissue-specific markers was detected by immunohistochemical staining with antibodies ag [file 2421689.f1.docx]

Coupled CRC 2D and ALI 3D Cultures Express Receptors of Emerging Viruses and Are More Suitable for the study of Viral Infections Compared to Conventional Cell Lines

**Siyu Xia^1#^, Jun Liu^1#^, Yan Yang^1^, Ming Wu^1^, Lina Ye^2^, Si Chen^1^, Tao Zhang^1^, Zhihong Zeng^2^, Kang Zhang^2^, Kaihong Cai^2^, Xiang Long^3^, Wenbin Gao^4^, Shisong Fang^5*^, Hui Li^1,*^**

^1^State Key Laboratory of Virology/Institute of Medical Virology, School of Basic Medical Sciences, Wuhan University, Wuhan, Hubei, 430071,China;

^2^Wuhan University Shenzhen Institute, Shenzhen, Guangdong,518057, China;

^3^ Peking University Shenzhen Hospital, Shenzhen, 518036,Guangdong;

^4^ Shenzhen Luohu People’s Hospital, Shenzhen, 518001,Guangdong;

^5^Shenzhen Center for Disease Control and Prevention, Shenzhen,Guangdong, 518055, China;

# These authors contributed equally to this work.

*Correspondence and reprint requests to

Dr. Hui Li, [somhli@whu.edu.cn](mailto:somhli@whu.edu.cn)

Dr. Shisong Fang, szcdcssfang@aliyun.com


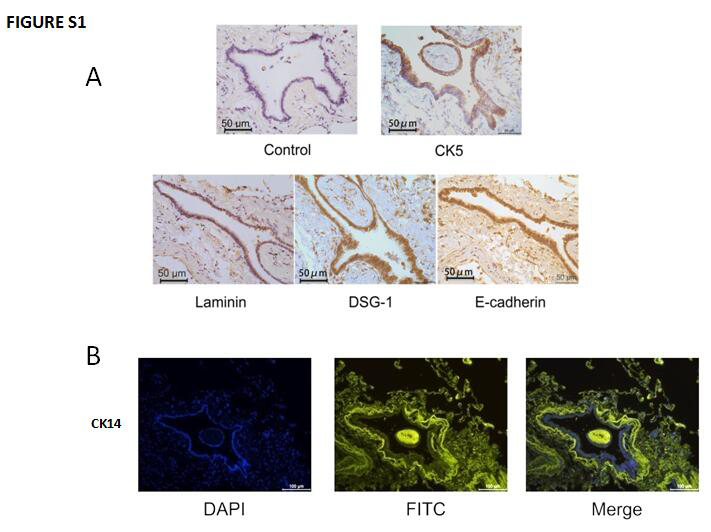


**Figure S1. Expression of tissue-specific markers in human tracheal tissue.** (A) Immunohistochemical staining of human tracheal tissue. Tissue was fixed by 4% paraformaldehyde (wt/vol), paraffin-embedded, sectioned detected by immunohistochemical staining with the specific antibodies against CK5, Laminin, DSG-1and E-cadherin. Scale bar, 50 μm. (B) Immunofluorensce staining of human tracheal tissue. Tissue was fixed by 4% paraformaldehyde (w/v), permeabilized with 0.5% Triton-X-100 and labeled the primary antibodies against CK14. The expression of CK14 was detected by immunofluorescence assay. The nuclei were stained by 0.5μg/ml DAPI. Magnification 20×.


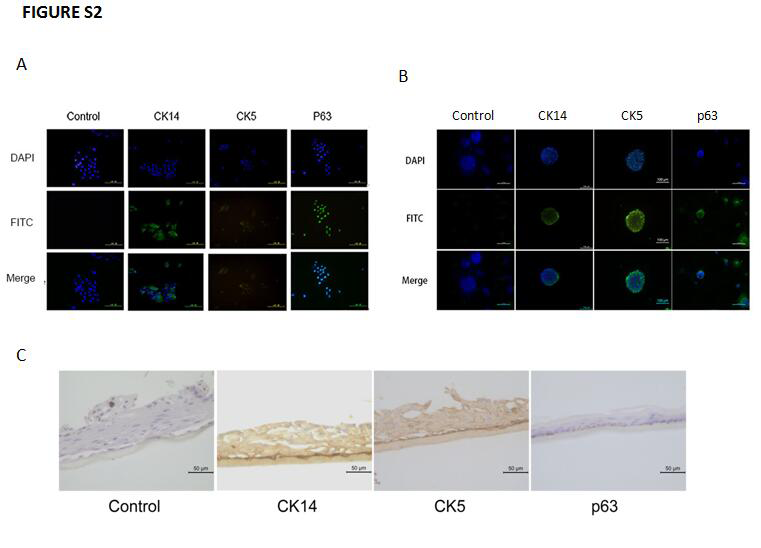


**Figure S2. Expression of tissue-specific markers in HNTEC cells and derived 3D cultures.** (A)Expression of tissue-specific markers in HNTEC cells. HNTEC cells were cultured on the sterile glass coverslips at an appropriate density and fixed in 4% (w/v) paraformaldehyde, permeabilized with 0.5% Triton-X-100 and labeled the primary antibodies against CK14, CK5 and p63 respectively. These three protein markers were detected by immunofluorescence assay. The nuclei were stained by 0.5μg/ml DAPI. The proteins were stained by second antibody goat-anti-mouse IgG—488. Scale bar, 100μm. (B) Expression of tissue-specific markers in matrigel 3D cultures of HNTEC cells. Single-cell suspension of HNTEC and A549 cells were cultured in medium containing 5% Matrigel for 7 days. Matrigel 3D cultures was fixed by 4% paraformaldehyde (w/v), permeabilized with 0.5% Triton-X-100 and labeled the primary antibodies against CK14, CK5 and p63. Scale bar, 100μm. (C) Expression of tissue-specific markers in ALI 3D cultures of HNTEC cells. HNTEC cells were cultured in air-liqiud interface (ALI) for 19 days and fixed by 4% paraformaldehyde (wt/vol), paraffin-embedded and sectioned using standard histological procedures. Expression of tissue-specific markers were detected by immunohistochemical staining with antibody against CK14, CK5, p63. Scale bar, 50μm.


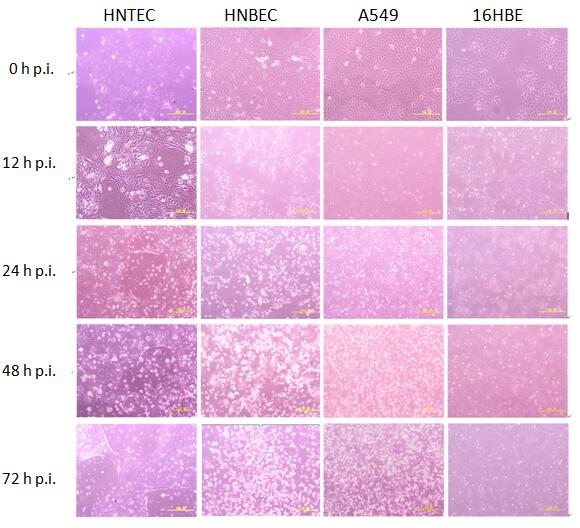


**Figure S3. H1N1 infection of 2D cultured airway normal epithelial cells.** HNTEC and HNBEC were seeded at 5x105per well in 6 well plates 24hrs before inoculation of H1N1pdm virus. A549 and 16HBE cells were as control cells. Morphology of cells was photographed at the indicated time points. Magnification 20×. Scale bar, 100μm.
